# Implementation Evaluation of a Complex Intervention to Improve Timeliness of Care for Veterans with Transient Ischemic Attack

**DOI:** 10.1007/s11606-020-06100-w

**Published:** 2020-11-03

**Authors:** T. M. Damush, E. J. Miech, N. A. Rattray, B. Homoya, Lauren S. Penney, A. Cheatham, S. Baird, J Myers, C. Austin, L J Myers, A J Perkins, Y. Zhang, B. Giacherio, M Kumar, LD Murphy, J J. Sico, D. M. Bravata

**Affiliations:** 1 Department of Veterans Affairs (VA) Health Services Research and Development (HSR&D) Precision Monitoring to Transform Care (PRIS-M) Quality Enhancement Research Initiative (QUERI), Indianapolis, IN USA; 2 VA HSR&D Center for Health Information and Communication (CHIC); Richard L. Roudebush VA Medical Center, 11H, 1481 W 10th St, Indianapolis, IN 46202 USA; 3 Department of Internal Medicine, Indiana University School of Medicine, Indianapolis, IN USA; 4 Regenstrief Institute, Indianapolis, IN USA; 5 South Texas Veterans Health Care System (STVHCS), San Antonio, TX USA; 6 University of Texas Health San Antonio, San Antonio, TX USA; 7 Department of Biostatistics, Indiana University School of Medicine, Indianapolis, IN USA; 8 Office of Healthcare Transformation (OHT), Veterans Health Administration (VHA), Washington, DC 20571 USA; 9 Clinical Epidemiology Research Center, VA Connecticut Healthcare System, West Haven, CT USA; 10 Departments of Internal Medicine and Neurology and Center for Neuro Epidemiological and Clinical Research, Yale School of Medicine, New Haven, CT USA; 11 Department of Neurology, Indiana University School of Medicine, Indianapolis, IN USA

**Keywords:** quality of care; implementation science; implementation strategy; audit and feedback; CFIR; transient ischemic attack; mixed methods

## Abstract

**Background:**

The Protocol-guided Rapid Evaluation of Veterans Experiencing New Transient Neurologic Symptoms (PREVENT) program was designed to address systemic barriers to providing timely guideline-concordant care for patients with transient ischemic attack (TIA).

**Objective:**

We evaluated an implementation bundle used to promote local adaptation and adoption of a multi-component, complex quality improvement (QI) intervention to improve the quality of TIA care Bravata et al. (BMC Neurology 19:294, 2019).

**Design:**

A stepped-wedge implementation trial with six geographically diverse sites.

**Participants:**

The six facility QI teams were multi-disciplinary, clinical staff.

**Interventions:**

PREVENT employed a bundle of key implementation strategies: team activation; external facilitation; and a community of practice. This strategy bundle had direct ties to four constructs from the Consolidated Framework for Implementation Research (CFIR): Champions, Reflecting & Evaluating, Planning, and Goals & Feedback.

**Main Measures:**

Using a mixed-methods approach guided by the CFIR and data matrix analyses, we evaluated the degree to which implementation success and clinical improvement were associated with implementation strategies. The primary outcomes were the number of completed implementation activities, the level of team organization and > 15 points improvement in the Without Fail Rate (WFR) over 1 year.

**Key Results:**

Facility QI teams actively engaged in the implementation strategies with high utilization. Facilities with the greatest implementation success were those with central champions whose teams engaged in planning and goal setting, and regularly reflected upon their quality data and evaluated their progress against their QI plan. The strong presence of effective champions acted as a pre-condition for the strong presence of Reflecting & Evaluating, Goals & Feedback, and Planning (rather than the other way around), helping to explain how champions at the +2 level influenced ongoing implementation.

**Conclusions:**

The CFIR-guided bundle of implementation strategies facilitated the local implementation of the PREVENT QI program and was associated with clinical improvement in the national VA healthcare system.

Trial registration: clinicaltrials.gov: NCT02769338

**Electronic supplementary material:**

The online version of this article (10.1007/s11606-020-06100-w) contains supplementary material, which is available to authorized users.

## BACKGROUND

Approximately 3300 veterans with transient ischemic attack (TIA) are cared for in a United States Department of Veterans Affairs (VA) Emergency Department (ED) or inpatient ward annually.^1^ TIA patients are at high risk of recurrent vascular events^2–4^; however, interventions which deliver timely TIA care can reduce that risk by up to 70%.^5–8^ Despite the known benefits of timely TIA care, gaps in care quality exist in both private-sector US hospitals^9^ and the VA healthcare system.^10^ A formative evaluation of TIA acute care in VA indicated that most facilities lacked a TIA-specific protocol and that clinicians struggled with uncertainty regarding the decisions to admit TIA patients for timely care.^11, 12^

The objective of the PREVENT trial^13^ was to evaluate the effectiveness of an implementation strategy bundle to promote local adaptation and adoption of a multi-component QI intervention to improve TIA care quality. The bundle was based upon several implementation frameworks and our previous success employing external facilitation with VA clinical teams.^14^ First, we used the integrated Promoting Action on Research Implementation in Health Services (iPARIHS) framework^15^ to guide external facilitation to assist local champions cultivate clinical teams, and disseminate professional education materials related to acute TIA care, and to facilitate local QI efforts using performance data. Second, based on prior studies which have distinguished CFIR constructs between high and low effective implementation,^16^ we operationalized key constructs (Planning; Goals & Feedback; and Reflecting & Evaluating) from the CFIR inner setting and implementation process domains^17^ a priori. Participating teams were trained on these concepts and received reinforcement through external facilitation.

The specific aim of this evaluation was to examine the effect of the implementation strategy bundle on implementation success. We hypothesized that clinical teams which engaged in the implementation strategies and locally adapted the PREVENT program components would realize the greatest implementation success.

## METHODS

### Setting

VA facilities were rank ordered in terms of the quality of TIA care based on seven guideline-concordant processes of care and invitations to participate were sent to VA facilities with the greatest opportunity for improvement. Recruitment stratified by region continued until six facilities agreed to participate. PREVENT sites were pragmatically allocated to the stepped-wedge trial in three waves based on the ability to schedule baseline and kickoff meetings.

### Quality Improvement Intervention

The rationale and methods used for the development of the PREVENT intervention have been described elsewhere.^13^ The provider-facing QI intervention was based on a prior systematic assessment of TIA care performance at VA facilities nationwide as well as an evaluation of barriers and facilitators of TIA care performance using four sources of information: baseline quality of care data,^10^ staff interviews,^11^ existing literature,^18–22^ and validated electronic quality measures.^10^ The PREVENT QI intervention included five components (see Appendix 1): quality of care reporting system (see Appendix 2), clinical programs, professional education, electronic health record tools, and QI support including a virtual collaborative.

### Implementation Strategies

PREVENT employed a bundle of three primary implementation strategies: (1) team activation via audit and feedback,^23, 24^ reflecting and evaluating, planning, and goal setting^17^; (2) external facilitation (EF)^23–25^; and (3) building a community of practice (CoP).^26^ In addition, PREVENT allowed for local adaptation of its intervention components and the coordinating site provided EF to the site champion and team.

Active implementation of PREVENT at each site began with a full-day kickoff meeting facilitated by the coordinating site, and involved multidisciplinary staff members from the participating site involved in TIA clinical care. The site team used the PREVENT Hub, a Web-based audit and feedback platform (see Appendix 2), to explore their facility-specific quality of care data and identify gaps. Using approaches from systems redesign,^27, 28^ site team members brainstormed about barriers to providing highest quality of care, identified solutions to address barriers, ranked solutions on an impact-effort matrix, and developed a site-specific action plan that included high-impact/low-effort activities in the short-term plan and high-impact/high-effort activities in the long-term plan. Local QI plans were entered into the PREVENT Hub, and metrics were tracked on the Hub allowing teams to monitor performance over time. Using the Hub to observe other participating sites’ QI activities and performance, facility teams could learn which QI activities either did or did not improve metrics at peer sites. In addition, the coordinating team introduced strategies for activating teams: team planning, goal setting and feedback, and reflecting and evaluating using examples and data from past stroke QI teams and presenting a video of a VA stroke QI team in practice.

During the 1-year active implementation period, the site team members joined monthly PREVENT virtual collaborative conferences which served as a forum for sites to share progress on action plans, articulate goals for the next month, and review new evidence or tools. EF was provided by the PREVENT nurse trained in Lean Six Sigma methodology^28^ and quality management.

### Evaluation Approach

The stepped-wedge^29, 30^ implementation trial included six participating sites where active implementation was initiated in three waves, with two facilities per wave. The unit of analysis was the VA facility.

### Measurement

We employed a mixed-methods design to evaluate this complex implementation intervention with prospective data collection from multiple sources. Qualitative data sources included the following: semi-structured interviews, observations, field notes, and Fast Analysis and Synthesis Template (FAST) facilitation tracking.^31^ Interviews were conducted in-person during site visits or by telephone at baseline, and at 6 and 12 months after active implementation. Key stakeholders included staff involved in the delivery of TIA care, their managers, and facility leadership; we also accepted “snowball” referrals from key stakeholders. Upon receipt of verbal consent, interviews were audio-recorded. The audio-recordings were transcribed verbatim. Transcripts were de-identified and imported into Nvivo12 for data coding and analysis.^32^

Using a common codebook, two team members independently coded identical transcripts for the presence or absence of CFIR constructs as well as magnitude and valence for four CFIR implementation constructs (i.e., Goals & Feedback, Planning, Reflecting & Evaluating, and Champions). Valence (+ or −) was scored for each construct if it was present and influencing the implementation of PREVENT at that site.^16, 17^ Magnitude was scored as 2 if it had a strong influence on PREVENT implementation, 1 if it had a weak or moderate effect, and 0 if it had a neutral effect. The evaluation team conducted formal debriefings after each kickoff, site visit, and collaborative call. These observations were recorded and transcribed for analyses.

We also used the FAST template, which is a structured electronic log, as a rapid, systematic method for extracting key concepts across data sources including interviews, collaborative calls, and Hub utilization data.^31^ We adapted an external facilitator tracking sheet for prospective collection of the dose and contents of site-specific, external facilitation provided by the evaluation team to participating site teams.^25^

#### Facility Baseline Characteristics

The measure of quality of care was the “without-fail” rate (WFR), defined as the proportion of veterans with TIA who received all of the processes of care for which they were eligible from among seven processes of care (Table 1). The WFR was calculated at the facility level based on electronic health record data using validated algorithms.^10^ In addition to the baseline WFR for each facility, data from the Office of Productivity, Efficiency and Staffing (OPES) were obtained to classify neurology and emergency medicine staffing levels (https://reports.vssc.med.va.gov/ReportServer/Pages/ReportViewer.aspx?/OPES/WorkforceRpt/WorkforceReport&rs:Command=Render&rc:Parameters=True&Specialty=Internal%20Medicine&FiscalYear=FY%202018). We report the annual TIA volume at each site (seen in the ED and inpatient setting) and the proportion of patients who were admitted.

**Table 1:** Facility Baseline Characteristics

Facility	Baseline performance*		Proportion of patients seen by primary care within 30-days of TIA^§^	Team description (as of the end of active implementation)	Proportion of TIA patients who were admitted^†^	Staffing^‡^		Annual TIA patient volume	Geographic region
	Without Fail Rate	Number of processes below national average (among 7 processes)							
						Neurology	Emergency medicine		
A	16.3%	6	58.7%	Senior Emergency Medicine nurse clinical champion supported by Internal Medicine leadership; team activation at basic level	58.7%	3.12 (3.12)	6.20 (6.20)	46	Southeast
B	33.3%	3	72.2%	Vascular neurologist serving as director of stroke services; multiple engaged pharmacy, telehealth nursing, Emergency Medicine clinicians; team activation at basic level	55.6%	4.00 (3.94)	6.28 (6.28)	18	West
C	38.5%	2	76.9%	Senior neurologist serving as director of stroke services; engaged pharmacy, Chief of Neurology, and Emergency Medicine clinicians; team activation at basic level	84.6%	3.55 (3.27)	7.03 (7.03)	13	Northeast
D	38.7%	3	71.0%	Vascular neurologist serving as director of stroke services; senior VA Systems Redesign champion; engaged Emergency Medicine, and hospitalist clinicians; Supportive Chief of Pt Safety; team activation at basic level	93.5%	5.37 (5.31)	7.81 (7.81)	31	Southeast
E	50.0%	3	75.0%	Vascular neurologist serving as director of stroke services; multiple engaged pharmacy, Emergency Medicine, and hospitalist medicine clinicians; team activation at basic level	87.5%	6.39 (3.87)	7.59 (7.59)	24	Midwest
F	55.2%	1	36.7%	Existing stroke team led by vascular neurologist who was relatively new to the VA; very engaged Emergency Medicine, Pharmacy, Education clinicians, Supportive Chief of Neurology; team activation at basic level	90.0%	6.25 (4.28)	6.05 (6.00)	30	South

*The Without Fail Rate (*WFR*) is calculated at the facility level and is the proportion of patients who receive all processes care for which they are eligible among seven processes (brain imaging, carotid artery imaging, neurology consultation, hypertension control, anticoagulation for atrial fibrillation, antithrombotics, and high/moderate potency statins). The national average WFR in fiscal year 2017 was 34.3%

^**§**^*TIA* refers to transient ischemic attack. The national average (fiscal year 2017) for the proportion of TIA patients who were seen by primary care within 30 days of discharge from the index TIA event was 60.8%

†The national average (fiscal year 2017) for the proportion of TIA patients who were admitted to the hospital (as opposed to being discharged home from the Emergency Department) was 67.4%

‡The staffing refers to full-time employee equivalents (FTEE) which are the actual worked hours adjusted for clinical time spent in direct patient care

### Implementation Evaluation

Teams reported on implementation progress on a monthly basis during the virtual collaborative calls. Teams were encouraged to adapt PREVENT components best suited for their local context and addressed gaps in care; thus, a fidelity evaluation was not applicable. The first of three primary implementation outcomes included the facility team’s *number of implementation activities* (defined as an intentional activity planned to change practices; aligned with each site’s action plans (see Appendix 3) and completed during the 1-year active implementation period).^14^

The second outcome included *final level of team organization* (defined as the degree of team cohesion and Group Organization [GO Score])^16, 33^ for improving TIA care at the end of the 12-month active implementation period. The GO Score^16, 33^ is a measure of team activation on a 1–10 scale for improving TIA care based on specified practices (see Table 2). The evaluation team independently determined each site’s GO Score by discussing evidence from the study data sources during team meetings and then voting independently using a digital, real-time anonymous ballot until 80% agreement was reached. The rationale for using both implementation outcomes was that they measured two distinct but complementary aspects of implementation: number of activities completed is an overall measure of implementation action, whereas the GO Score describes the degree to which the facility team is functioning as a unit to implement facility-wide policies and structures of care.

**Table 2:** Implementation Strategies and Outcomes

WAVES	Facility	Implementation strategies							Prevent local adaptation						Implementation outcomes	
		External facilitation*					Community of practice attendance^§^	Hub^†^	PREVENT components local adaptation						Number of implementation activities completed	Final GO** Score
		Education	Quality process monitoring	Planning	Networking	Nurse EF episodes			Professional education	Data feedback	Quality improvement support	Clinical programs	EHR^‡^ tools	Other		
1	Site F: champion	10	9	6	8	21	10 of 12 (83%)	16 (1)	5	2	1	11	4	5	28	8
	All others						36/7	8 (4)								
	Site C champion	8	9	16	12	26	8 of 12 (66%)	8 (1)	3	6	1	5	3	1	19	7
	All others						25/5	9 (2)								
2	Site E: champion	12	18	17	18	35	10 of 12 (83%)	11 (1)	9	1	1	10	7	5	33	4
	All others						24/7	25 (5)								
	Site A: champion	6	9	8	7	19	12 of 13 (92%)	50 (1)	13	5	2	9	7	3	39	8
	All others						26/9	21 (11)								
3	Site B: champion	9	7	10	9	19	9 of 12 (75%)	23 (1)	7	2	1	5	5	5	25	7
	All others						13/6	33 (6)								
	Site D: champion	4	9	10	9	19	11 of 13 (84%)	20 (3)***	6	1	1	2	2	3	15	6
	All others						7/6	25 (9)								
AVERAGE		8.17	10.17	11.7	10.5	23.17	80.5%	21.3	7.17	2.83	1.17	7.00	4.00	3.67	26.50	6.67
								20.2								

*The dose of site-specific, external facilitation (*EF*) to the site-specific quality improvement teams was evaluated across distinct categories; the most common categories were education (educating the site QI team on PREVENT component), quality monitoring (assisting the site QI team with data interpretation), planning (assisting QI team with implementation plans), and networking (introductions across site QI teams). Dose was defined as EF interaction with site member by phone, email, Skype, or in person. See Appendix 1 for a complete description of the PREVENT components

^**§**^The teams joined monthly PREVENT collaborative conferences which served as a forum for facility team members to share progress on action plans, articulate goals for the next month, and review any new evidence or tools. Collaborative conference attendance includes the total number of calls that team members joined during the 1-year active implementation period plus the first month during the kickoff when the kickoff occurred early in the month for a total of 13 possible calls, as well as the number of unique team members who participated. After active implementation completion, participants were permitted to join the calls but no longer participated in updates. For example, at facility F, the 7 unique team members’ (excluding the champion) participation distribution was 36 call attendings over the 22 possible calls. Note, not all 7 team members participated in all 22 calls. Wave 1 sites had the opportunity to attend more collaborative conferences than wave 2 or wave 3 sites

†The Web-based PREVENT Hub provided performance data about a broad range of processes of care, healthcare utilization, and other aspects of care; these data were updated monthly for every VA facility. The Hub also displayed facility implementation plans and included a library of resources. Hub utilization is displayed in terms of the number of total visits by any team member from the facility during the 1-year active implementation period; the number of unique individuals is in parentheses

‡*EHR* refers to electronic health record

**The *GO* Sco*re* refers to the Group Organization Score for improving TIA care quality; it is a measure of team activation and cohesion. The GO Score is measured on a scale of 0–10 based on specific practices in place during a given time period and scored by the evaluation team. A score of 0–3 indicates the absence of a facility-wide approach; 4–5 reflects a developing facility-wide approach; 6–7 denotes basic proficiency with the presence of a comprehensive facility-wide program; and 8–10 indicates the presence of a mature, facility-wide system that can sustain key personnel turnover. Because the GO Score reflects team activation and cohesion, some sites with individuals who are actively engaged in quality improvement but without a robust team approach may have lower GO Scores but still complete a relatively high number of implementation activities

***Site D had a total of 3 local champions over the course of one year of active implementation. The first champion left the organization within the first 6 months of active implementation; the second champion emerged from the team and lead the transition; and the third champion joined the transitional champion to implement the action plans

As an additional clinical measure of implementation outcome, the final column in Table 3 indicates whether or not the facility achieved ≥ 15-point improvement (in absolute terms to reflect planned vs temporal change) in their WFR over their 1-year course of active implementation (see Table 1).

**Table 3:** Matrix Display of Longitudinal Implementation Data

Sites	CFIR construct scores*				Primary implementation outcomes				Clinical outcome
	Reflecting & Evaluating	Goals & Feedback	Planning	Champions	Number of implementation activities completed	Team activation GO^§^ score			15 points or greater improvement in Without Fail Rate over 1-year period of active implementation
						Baseline	Midpoint	Final	
Site F					28	1	7	8	Yes
6 months	+ 2	+ 2	+ 2	+ 2					
12 months	+ 1	+ 2	+ 2	+ 2					
Site A					39	1	6	8	Yes
6 months	+ 2	+ 2	+ 2	+ 2					
12 months	+ 2	+ 1	+ 2	+ 2					
Site C					19	1	7	7	Yes
6 months	+ 1	+ 1	0	+ 2					
12 months	0	0	0	+ 1					
Site B					25	1	5	7	No
6 months	+ 1	+ 1	+ 1	+ 1					
12 months	+ 2	+ 2	+ 2	+ 2					
Site D					15	1	4	6	No
6 months	**−** 1	**−** 1	**−** 1	**−** 1					
12 months	+ 1	+ 1	+ 1	+ 2					
Site E					33	1	4	4	Yes
6 months	0	0	+ 1	+ 1					
12 months	+ 1	+ 1	+ 1	+ 1					

Sites are presented in the order of their Final Team Activation GO Score from highest to lowest.

*CFIR refers to the Consolidated Framework Implementation Framework. The magnitude and valence for four selected CFIR implementation constructs (i.e., Goals & Feedback, Planning, Reflecting & Evaluating, and Champions) were coded for each site at 6 months and 12 months after the initiation of the 1-year active implementation period

^**§**^The *GO* Sco*re* refers to the Group Organization Score for improving TIA care quality; it is a measure of team activation and cohesion. The GO Score is measured on a scale of 0–10 based on specific practices in place during a given time period. A score of 0–3 indicates the absence of a facility-wide approach; 4–5 reflects a developing facility-wide approach; 6–7 denotes basic proficiency with the presence of a comprehensive facility-wide program; and 8–10 indicates the presence of a mature, facility-wide system that can sustain key personnel turnover. Because the GO Score reflects team activation and cohesion, some sites with individuals who are actively engaged in quality improvement but without a robust team approach may have lower GO Scores but still complete a relatively high numbers of implementation activities

Using a mixed-methods approach^34, 35^ grounded in the CFIR,^16, 17^ we conducted a cross-case and data matrix approach^36^ to evaluate the degree to which the sites engaged in the bundle of implementation strategies; the association between implementation strategies and implementation and clinical success; and the associated contextual factors. Given that PREVENT’s implementation strategies and outcomes were tracked and rated by the coordinating team prospectively, no data were missing.

## RESULTS

### Baseline Context

The baseline facility context and QI team characteristics of the six participating facilities are provided in Table 1, listed in order of their baseline quality (WFR). The WFR for sites B, C, and D was similar to the fiscal year 2017 national WFR average of 34.3%, whereas site A was substantially below and Sites E and F were considerably higher than the national WFR. Sites E and F also had the highest level of neurology staffing. Emergency medicine staffing was similar across sites. More than 50% of TIA patients were admitted to the hospital, but admission rates were lowest at the two sites (A and B) with the lowest WFRs. The annual TIA patient volume varied from 13 to 46. At baseline, no sites had active teams in place working on TIA care quality, indicating that all of the teams began the active implementation period from a similar starting point. The participating site QI teams were diverse but generally included members from neurology, emergency medicine, nursing, pharmacy, and radiology; some teams also included hospitalists, primary care staff, education staff, telehealth staff, or systems redesign staff.

### Implementation Strategies

Over the course of the 1-year active implementation period, we observed an overall high site engagement with each of the implementation strategies. In Table 2, we present the dose of implementation strategies delivered within the overall strategy bundle: EF, community of practice, Hub (audit and feedback), and local adaptation of PREVENT. The total number of completed implementation activities and final GO Score after 12 months of active implementation are also presented in Table 2. Site labels are retained from Table 1 and are listed in order of the three waves.

### Audit and Feedback

We observed frequent usage of the Hub. The quality of care data (i.e., the 7 processes of care that comprised WFR) was updated monthly on the Hub; the average site champion Hub usage was 21.3 visits per 12 months (1.8 visits per month) and the average non-champion team member Hub usage was 20.2 visits per 12 months (1.7 visits per month). This Hub usage aligns with interview data from site team members indicating that they used the Hub for the process of care data, to access the QI plans, and to download materials from the library:

I [champion] used some of those slides [hub library] in order to show them [providers at local site] what the PREVENT program was and why it’s important.

### External Facilitation

Facility QI teams and champions engaged with the EF during active implementation. Education on PREVENT components during the first half of the year, quality monitoring, planning, and networking between and within sites were the most commonly employed EF topics. Overcoming barriers and data reflection and evaluation were also frequent EF tasks.

The EF was really helpful in allowing me to …call and vent, and she was also really very encouraging. …That was an interesting lesson to learn that you might feel like you’re unsuccessful because of that one particular metric, …and so I appreciate her lending me her ear...

When the [EF] knew that I was encountering a barrier that was related to physicians, without asking, she would immediately provide me the data that I needed to discuss with that provider to make them understand what we were doing and that was really helpful.

### Community of Practice

Sites were active participants in the virtual CoP. A site representative was present on all calls. Site champions attended an average of 80.5% of the monthly collaborative conferences (range 66 to 92%) during the active implementation period. All six teams participated in a promotion ceremony which was attended by local VA facility leadership as well as all of the PREVENT sites’ team members; peers from other sites acknowledged the implementation successes and lessons learned from the graduating site.

### Implementation Outcomes

The implementation outcome data (Tables 2 and 3) indicated that implementation took place at all facilities given that all sites successfully completed at least 15 implementation activities (range 15–39, mean 26.5) as part of their action plans to improve the quality of TIA care over 12 months. Despite heavy clinical demands at all participating sites, none of the teams withdrew from PREVENT.

Table 3 provides longitudinal data for implementation outcomes as well as scores on CFIR constructs related to the implementation strategies which PREVENT facilitated among the local clinical champions and QI teams during the year of active implementation. The sites were ranked in terms of the final GO Score. All sites achieved a substantial increase in the GO Score from baseline to the midpoint of the active implementation period (6 months): mean of 1 at baseline (no facility-wide approach) to 5.5 at 6 months (some facility-wide approach activities). All sites began at the same level, 1, because they had no pre-existing organization around TIA. The kickoff and the Hub allowed teams to examine their data, identify gaps in care, develop an action plan, and start to come together as a team; these two elements of the PREVENT intervention were the key factors responsible for the observed improvements in team activation across sites during the first 6 months of active implementation.

I came out of it [kickoff] feeling that I knew what the issue is…I know what the goal is, and I have information sources so that I'm able to do it. …. one of the biggest things that I see is that I think that it really helped to come up with like more of a team…

I think [the kickoff] played a large role of how we decided we wanted to proceed … I feel like that was the first time we’ve, we really kind of drilled down on, on a good …starting plan …of an outline of what we wanted to accomplish.

The matrix display in Table 3 also indicates the key role of champions in promoting implementation success, as there was a direct link between the strong presence of effective champions (i.e., “+2” level) and the GO Score at both the 6- and 12-month timepoints. At 6 months, the correspondence between a champion score of + 2 and a GO Score of ≥ 6 (cf. sites F, A, and C) was 100% (3/3). None of the other CFIR constructs systematically distinguished the three sites with scores ≥ 6 from the three sites without. Moreover, all three of these sites with GO Scores ≥ 6 at the 6-month timepoint ultimately achieved ≥ 15 point improvements (in absolute terms) in their WFR rates over the 1-year active implementation period, explaining 75%, 3 of the 4 sites, with ≥ 15 point gains.

At 12 months, the correspondence between a champion score of + 2 and a positive gain in the GO Score between the 6- and 12-month timepoints was likewise 100% (cf. sites F, A, B, D). As before, none of the other constructs systematically distinguished the four sites that improved their GO Score between the 6- and 12-month timepoints from those that did not.

Furthermore, Table 3 indicates that the presence of an effective champion was a necessary but not sufficient condition for the strong presence of the CFIR constructs of Reflecting & Evaluating, Goals & Feedback, and Planning. For all five of the rows in Table 3, where a + 2 score appeared for any of these three CFIR constructs (cf. site F, 6 and 12 months; site A, 6 and 12 months; site B, 12 months), the correspondence with a champion score of + 2 was 100% (5/5). The reverse was not true, however: the correspondence between sites with + 2 scores for champions with + 2 scores for all 3 CFIR constructs of was only 43% (3/7) (cf. site A, 12 months, Reflecting & Evaluating = + 1; site C, 6 months, Planning = 0; site D, 12 months, all 3 constructs = +1). This indicates that the strong presence of effective champions acted as a pre-condition for the strong presence of Reflecting & Evaluating, Goals & Feedback, and Planning (rather than the other way around), helping to explain how champions at the + 2 level influenced ongoing implementation.

Site E had multiple individuals who engaged in clinical champion activities, and therefore had lower champion construct scores and achieved lower GO Scores. For example, individual team members at site E often engaged in activities that addressed processes within their own service areas, rather than carrying out coordinated, cross-service efforts led by a central champion. The negative valence in site D’s clinical champion construct reflected turnover in their local champion early in active implementation which resulted in the lowest total number of activities completed. However, with the replacement of the local champion, addition of new team members, dedicated EF, and CoP and Hub engagement by the new champion and team members, site D’s GO Score improved.

**Figure 1:**
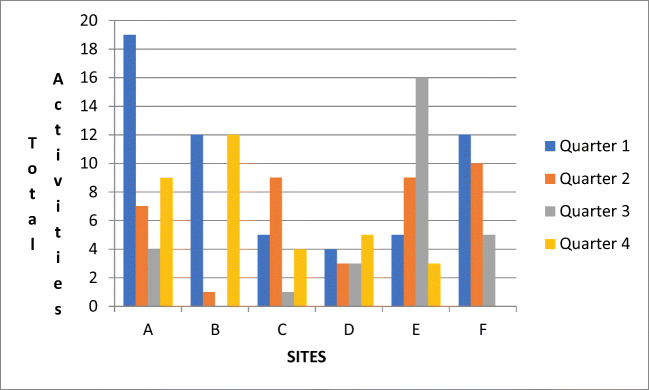
Implementation activities over the 1-year active implementation period.

Although the sites completed a diverse range of implementation activities (Table 2), the most common categories included activities related to professional education (e.g., teaching house staff) and implementation of clinical programs (e.g., prospective patient identification systems). Teams that engaged in planning to the greatest degree were those with the highest number of completed implementation activities (Table 3). Sites varied considerably in terms of the timing of the implementation activities completed over the 1 year of active implementation (Fig. 1, Appendix 3). Half of the teams began strongly with implementation soon after the kickoff whereas other teams engaged in more activities during the latter half of the year.

## DISCUSSION

Our data suggested that the presence of an effective champion was key for implementation success . Indeed, when one site lost its champion, implementation progress was halted, and then revived with the replacement of the local champion who was subsequently supported with EF. Effective champions, individuals who “drive through” implementation according to CFIR^16^, appear to play a critical role in engaging in the implementation strategies including the EF, and fostering teams in reflecting and evaluating, goal setting, and planning: activities which were related both to the total number of implementation activities completed and the degree of team cohesion.^37, 38^

Our results also suggest that an alternative approach to implementation occurred at a site with a distributed champion model—one in which several individuals shared the responsibility for actions usually performed by a champion. In this context, the individuals limit the activities in which they drive through implementation to their specific clinical area versus the overall program. Although this site (E) did not achieve high scores for team functioning, they did complete many implementation activities. A key to the success of this approach is the degree to which the various champions are able to independently complete a given implementation activity with limited guidance by a central champion

PREVENT site champions were diverse, including staff from neurology, nursing, pharmacy, and systems redesign. Our study findings indicated that the professional discipline of the champion was less important than the role they played in either performing implementation activities themselves or engaging other team members in the QI process.

The site team members, and especially the champions, regularly contacted the EF who provided information, support, and encouragement across a broad range of topics. Two key EF activities merit further description: conducting chart reviews and facilitating implementation of the patient identification tool. Clinical champions were sometimes dismayed when the monthly performance data indicated that some patients had failed process measures. The EF conducted targeted chart review which identified gaps in care or documentation; this chart review information supported the champions in their efforts to engage in quality improvement. The EF also worked with teams to implement a patient identification tool to identify patients with TIA who were cared for in the ED or inpatient setting. This tool was used at some sites to prospectively ensure that patients received needed elements of care and at other sites to retrospectively identify opportunities for improvement. Given that many of the champions were clinicians without prior QI experience, the EF was able to help connect clinicians with local clinical informatics staff to implement the patient identification tool.

These findings have direct relevance for healthcare systems like the VA where quality improvement resources may need to be targeted at lower performing facilities which may lack existing teams, have small patient volume, and vary considerably in terms of baseline context. The current findings emphasized the importance of EF and indicate that EFs should be flexible given the heterogeneity in site needs. Moreover, the combination of the in-person kickoff meeting and the Hub at the launch of the active implementation period was critical in three domains/areas: the development of site-specific action plans that were based on site-specific performance data; the early formation of team identity; and to the training of champions and QI teams on how to reflect and evaluate on their data. The high degree of Hub usage suggests that healthcare systems implementing QI programs should provide a forum for providing updated site-level quality of care data as well as resources for quality improvement that can be readily shared across sites. Finally, healthcare systems should consider supporting QI teams within CoP that serve as supported arenas for public accountability for making progress on action plans, sharing lessons learned, and providing encouragement.

### Contribution to the Literature

To our knowledge, the PREVENT implementation strategy bundle is one of the few implementation interventions to operationalize CFIR implementation process constructs as strategies a priori to train its QI teams and prospectively evaluate its uptake. Indeed, a recent review of CFIR usage in implementation research recommended future research identified the need for research with prospective CFIR use with a priori specification.^34^ Moreover, we provided specifications^39^ on the set of implementation strategies delivered as a bundle. In addition, we described the level and timing of engagement and implementation activities across QI teams. These findings provide evidence for specific implementation strategies in the setting of a complex clinical problem when no quality of care reporting system exists.^40^

Our results are aligned with a recent systematic review of the effect of clinical champions on implementation which concluded champions were necessary but insufficient for implementation.^41^ An emergent finding was that modest and strong positive CFIR planning construct was related to implementation success (especially with respect to the number of implementation activities completed).

### Limitations

The primary limitation of PREVENT is that implementation occurred only within VA hospitals. Future research should evaluate implementation in non-VA settings. Because several implementation strategies were deployed simultaneously, it was difficult to disentangle the unique effects of each strategy. Although a six-site sample was sufficient to make case comparisons, future studies might include a larger number of facilities to evaluate additional implementation outcomes.

In summary, this study found that engagement in a bundle of CFIR-related implementation strategies facilitated the local adaptation and adoption of the PREVENT TIA QI program and that two alternative approaches to the role of champions were associated with implementation success.

## Electronic supplementary material

ESM 1 (DOCX 224 kb)

## Data Availability

These data must remain on Department of Veterans Affairs servers; investigators interested in working with these data are encouraged to contact the corresponding author.

## **ABBREVIATIONS**

ED: Emergency Department
PREVENT: Protocol-guided Rapid Evaluation of Veterans Experiencing New Transient Neurological Symptoms
QI: quality improvement
TIA: Transient ischemic attack
VA: Department of Veterans Affairs
EF: External Facilitation or External Facilitator
WFR: Without Fail Rate
EHR: Electronic Health Record
GO Score: Group Organization Score
CFIR: Consolidated Framework for Implementation Research
CoP: Community of Practice
