# Supplementary material for: Implementation Evaluation of a Complex Intervention to Improve Timeliness of Care for Veterans with Transient Ischemic Attack
Source: J Gen Intern Med. 2020 Nov 3;36(2):322–32. doi: 10.1007/s11606-020-06100-w (PMC7878645; doi:10.1007/s11606-020-06100-w)
Supplement: Supplementary file 1 — (DOCX 224 kb) [file 11606_2020_6100_MOESM1_ESM.docx]

**SUPPLEMENTARY MATERIALS**

**Appendix A. Local PREVENT Quality Improvement Intervention Components designed to overcome systemic barriers to acute TIA care.**

**
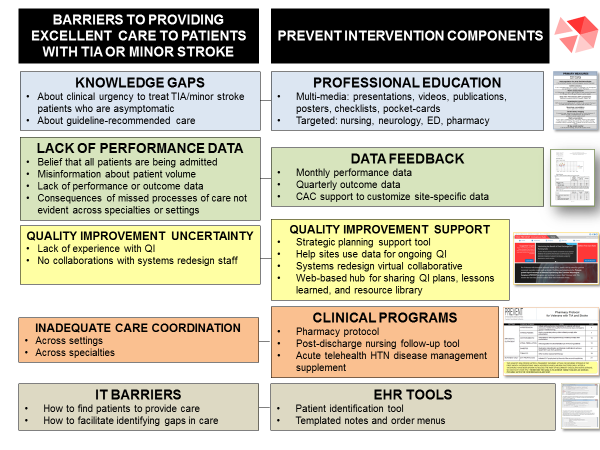
**


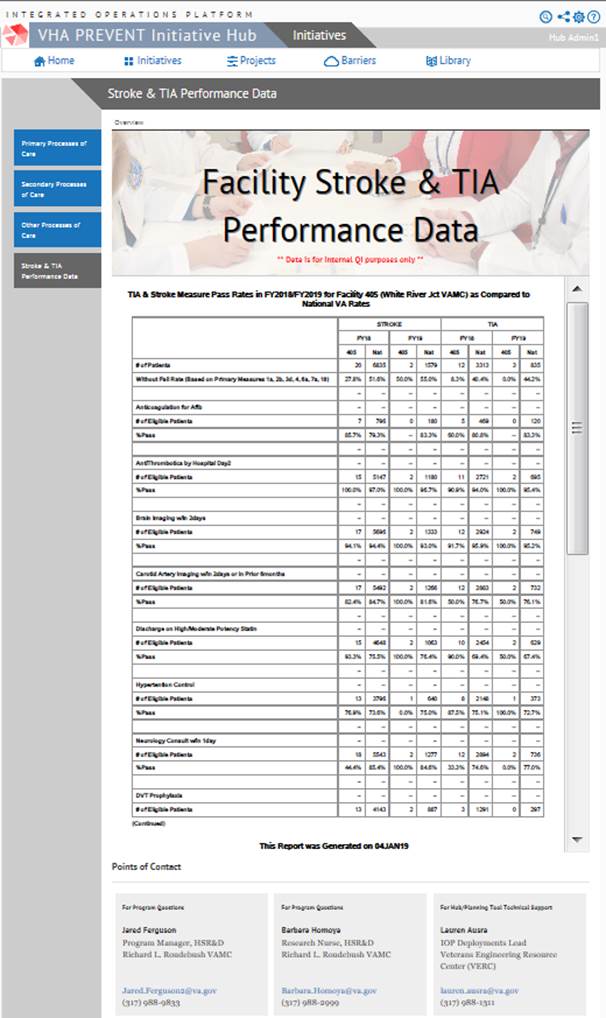


Appendix B: PREVENT Integrated Operations Platform (IOP) Data Hub

Screen Shot

**Appendix C. Local Adaptation and Implementation Activities of Prevent Components over Time**

| **Site A** | **Quarter 1** | **Quarter 2** | **Quarter 3** | **Quarter 4** | | **TOTAL** |
| --- | --- | --- | --- | --- | --- | --- |
| Professional Education | 9 | 1 |  | 3 | | 13 |
| Data Feedback | 1 | 1 | 1 | 2 | | 5 |
| Quality Improvement Support | 2 |  |  |  | | 2 |
| Clinical Programs | 3 | 3 | 1 | 2 | | 9 |
| E H R Tools | 3 | 2 | 2 |  | | 7 |
| Other | 1 |  |  | 2 | | 3 |
| TOTAL | 19 | 7 | 4 | 9 | | 39 |
| **Site B** | **Quarter 1** | **Quarter 2** | **Quarter 3** | **Quarter 4** | | **TOTAL** |
| Professional Education | 6 | 1 |  |  | | 7 |
| Data Feedback | 1 |  |  | 1 | | 2 |
| Quality Improvement Support | 1 |  |  |  | | 1 |
| Clinical Programs | 2 |  |  | 3 | | 5 |
| E H R Tools | 2 |  |  | 3 | | 5 |
| Other |  |  |  | 5 | | 5 |
| TOTAL | 12 | 1 | 0 | 12 | | 25 |
| **Site C** | **Quarter 1** | **Quarter 2** | **Quarter 3** | **Quarter 4** | | **TOTAL** |
| Professional Education | 1 | 1 |  | 1 | | 3 |
| Data Feedback | 1 | 5 |  |  | | 6 |
| Quality Improvement Support | 1 |  |  |  | | 1 |
| Clinical Programs |  | 2 |  | 3 | | 5 |
| E H R Tools | 1 | 1 | 1 |  | | 3 |
| Other | 1 |  |  |  | | 1 |
| TOTAL | 5 | 9 | 1 | 4 | | 19 |
| **Site D** | **Quarter 1** | **Quarter 2** | **Quarter 3** | **Quarter 4** | | **TOTAL** |
| Professional Education | 2 | 1 | 1 | 1 | | 6 |
| Data Feedback |  |  | 1 |  | | 1 |
| Quality Improvement Support |  |  | 1 |  | | 1 |
| Clinical Programs |  | 1 |  | 1 | | 2 |
| E H R Tools |  |  |  | 2 | | 2 |
| Other | 2 | 1 |  |  | | 3 |
| TOTAL | 4 | 3 | 3 | 5 | | 15 |
| **Site E** | **Quarter 1** | **Quarter 2** | **Quarter 3** | **Quarter 4** | | **TOTAL** |
| Professional Education | 1 | 2 | 4 | 2 | | 9 |
| Data Feedback |  | 1 |  |  | | 1 |
| Quality Improvement Support |  | 1 |  |  | | 1 |
| Clinical Programs | 2 | 1 | 6 | 1 | | 10 |
| E H R Tools | 2 | 2 | 3 |  | | 7 |
| Other |  | 2 | 3 |  | | 5 |
| TOTAL | 5 | 9 | 16 | 3 | | 33 |
| **Site F** | **Quarter 1** | **Quarter 2** | **Quarter 3** | | **Quarter 4** | **TOTAL** |
| Professional Education | 3 | 2 |  | |  | 5 |
| Data Feedback | 1 |  | 1 | |  | 2 |
| Quality Improvement Support | 1 |  |  | |  | 1 |
| Clinical Programs | 4 | 6 | 2 | |  | 11 |
| E H R Tools | 1 | 2 | 1 | |  | 4 |
| Other | 4 |  | 1 | |  | 5 |
| TOTAL | 13 | 10 | 5 | | 0 | 28 |

**Note:** All six sites used all 5 components of the PREVENT QI program; however, each site had the opportunity to locally adapt the components which best suited their local context. Implementation activities were prospectively recorded by the evaluation team and totaled.

**Examples of site implementation activities as local adaptions of each of the five PREVENT Components**

**Professional Education**: Used PREVENT’s standardized educational materials including power point slides and targeted a variety of clinical providers in the Emergency Department, Pharmacy, Residents, Hospitalists, Neurology, Nursing. Each site chose how often, when, the contents and with whom to which they implemented Professional Education.

**Data Feedback:** Emergency medicine clinician from local QI team sent email with feedback of WFR to local ED staff congratulating them on their improved quality and encouraging sustained efforts.

**Quality Improvement Suppor**t: Service chiefs adopted the local PREVENT QI program as their annual pay for performance on innovation metric during the 1 year of active implementation.

**Clinical Programs**: Local champion conducted rounds with clinical providers for all TIA patients using a PREVENT without fail checklist.

**E H R Tools**: Site integrated ABCD2 score into local EHR for use in evaluating TIA patients.

**OTHER**: Asked a colleague associated with the VA regional unit to propose the local team’s dashboard request, received approval, and a regional cerebrovascular dashboard was built and used daily.
